# Nanoparticles for imaging-guided photothermal therapy of colorectal cancer

**DOI:** 10.1016/j.heliyon.2023.e21334

**Published:** 2023-10-20

**Authors:** Omid Rahbar Farzam, Niloofar Mehran, Farzaneh Bilan, Ehsan Aghajani, Reza Dabbaghipour, Ghazale Asemani Shahgoli, Behzad Baradaran

**Affiliations:** a Immunology Research Center, Tabriz University of Medical Sciences, Tabriz, Iran; b Department of Medical Biotechnology, School of Medicine, Kermanshah University of Medical Sciences, Kermanshah, Iran; c Department of Medical Genetics, Shiraz University of Medical Sciences, Shiraz, Iran; d Faculty of Medicine, Tabriz University of Medical Sciences, Tabriz, Iran; e Clinical Research Development Unit, Imam Reza General Hospital, Tabriz University of Medical Sciences, Tabriz, Iran

**Keywords:** Colorectal cancer, Nanoparticles, Photothermal therapy, PTT

## Abstract

Colorectal cancer (CRC) is one of the most common malignancies with a high mortality rate worldwide. While surgery, chemotherapy, and radiotherapy have shown some effectiveness in improving survival rates, they come with drawbacks such as side effects and harm to healthy tissues. The theranostic approach, which integrates the processes of cancer diagnosis and treatment, can minimize biological side effects. Photothermal therapy (PTT) is an emerging treatment method that usages light-sensitive agents to generate heat at the tumor site and induce thermal erosion. The development of nanotechnology for CRC treatment using imaging-guided PTT has garnered significant. Nanoparticles with suitable physical and chemical properties can enhance the efficiency of cancer diagnosis and PTT. This approach enables the monitoring of cancer treatment progress and safeguards healthy tissues. In this article, we concisely introduce the application of metal nanoparticles, polymeric nanoparticles, and carbon nanoparticles in imaging-guided PTT of colorectal cancer.

## 1 Introduction

Colorectal cancer (CRC) is a significant public health issue, ranking as the third most prevalent cancer worldwide and one of the most lethal [1]. The occurrence and mortality of this disease increase with age, and in developed countries, the average age of diagnosis is nearly 70 years [2]. Apart from age, various factors contribute to the development of CRC, including a history of colon polyps, diabetes mellitus, inflammatory bowel diseases or cancer, and unhealthy lifestyle choices such as obesity, alcohol consumption, smoking, and poor dietary habits [3]. CRC mostly includes the colon and rectal adenocarcinoma, which is considered the transformation of the normal epithelium into a precancerous lesion and finally into an invasive carcinoma [4]. This process requires the gradual accumulation of a series of genetic and epigenetic changes. Some of the most significant epigenetic changes studied in CRC are histone changes, DNA methylation, and changes in the expression of non-coding RNAs [5].

Metastasis and the spread of CRC to various distant organs is one of the problems in treating patients with CRC. Currently, common treatments for CRC include surgery, chemotherapy, and radiotherapy [6]. Surgery is typically the primary approach for patients with CRC in stages I to III. However, a considerable number of patients diagnosed in the early stages experience disease recurrence following surgical removal. Additionally, metastatic disease often develops, primarily affecting the lungs or liver. When feasible, chemotherapy is frequently employed as an adjuvant therapy to mitigate the risk of recurrence [7]. Oxaliplatin and 5-fluorouracil (5-FU) are the first-line drugs for CRC chemotherapy and are widely used [8]. But resistance to chemotherapy drugs is one of the main challenges of this treatment method, which mainly causes tumor recurrence, metastasis, and poor prognosis in patients. Changes in the target sites of chemotherapy agents, inhibition of tumor apoptosis, heterotrophy of tumor cells and cancer stem cells can lead to resistance to chemotherapy. Among these factors, cancer stem cells play a pivotal role in chemotherapy resistance, as they cannot be entirely eradicated during treatment and are the primary cause of tumor recurrence [9]. Radiotherapy, which is used as a definitive or adjuvant method for CRC, in addition to the acute toxicity of this treatment method, causes late side effects due to the functional sensitivity of the relevant structures [10]. Overall, the 5-year survival rate for patients with metastatic CRC is a mere 14 %, even after undergoing surgery, chemotherapy, radiotherapy, and other treatments. Consequently, it is imperative to explore novel and effective therapeutic strategies for the management of CRC [6].

**Fig. 1:**
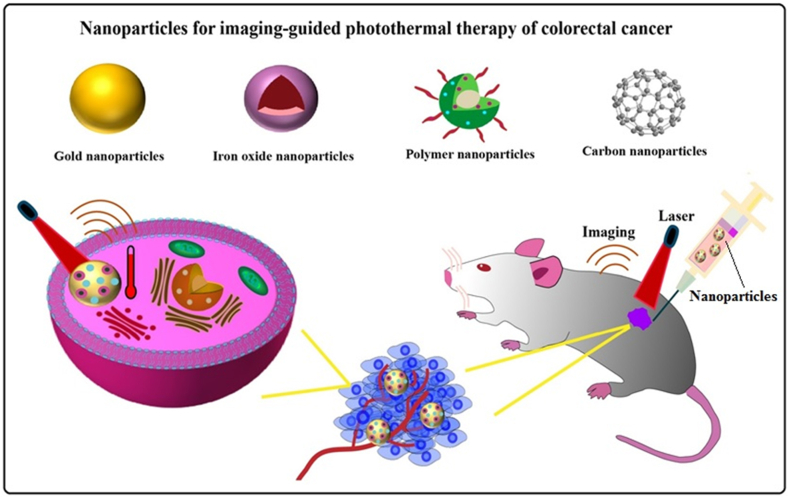
Schematic illustration of the application of nanoparticles in imaging-guided PTT in the treatment of colorectal cancer.

Cancer theranostics is a combination of cancer diagnosis and treatment methods, the goal of which is early diagnosis, imaging, and accurate treatment at the right time and dose, followed by monitoring the effectiveness of the treatment [11]. Various non-invasive imaging methods, such as Magnetic Resonance Imaging (MRI), Computed Tomography (CT), Diffusion Weighted Imaging (DWI), Positron Emission Tomography (PET) etc., can be commonly used in the diagnosis of CRC [12]. Light-based platforms can be utilized to optimize treatment outcomes in the field of cancer theranostics. It has been demonstrated that the use of light to induce heat, in addition to inducing tumor hyperthermia, also facilitates the rapid diffusion of therapeutic compounds at the targeted site [13]. Photothermal therapy (PTT) is one of the tumor phototherapy methods. In this method, photosensitive agents provide local destruction of cancer cells by absorbing laser energy in the near-infrared (NIR) region and converting it into heat [14]. In recent years, the advancements in nanoparticles in the field of biomedicine, such as PTT, have garnered the attention of researchers. These nanoparticles possess biocompatibility and optical absorption properties, making them highly valuable in PTT applications and effective photothermal converters. Their small size, low toxicity, and controlled drug release provide them with potential advantages. Furthermore, the modification of various nanostructures can alter their biological stability and enhance the toxicological concerns associated with certain nanostructures [15,16]. Therefore, this article aims to evaluate several nanoparticles utilized in imaging-guided PTT for CRC (Fig. 1).

## 2 Targeting techniques achieved for colorectal cancer

Although metastatic CRC remains challenging, a better understanding of the pathways involved in cancer cell proliferation and evolution has led to the development of targeted therapies. Targeted therapies that focus on specific biological characteristics of cancer cells target cellular pathways involved in cancer development [17]. This new treatment method has been able to increase the overall survival of patients with CRC. Following the successes achieved with the anti-angiogenic agent bevacizumab and the anti-epidermal growth factor receptor (EGFR) agent cetuximab, new agents that block immune checkpoints and various critical pathways are rapidly emerging [18]. Several targeted drugs that have been developed and studied, by targeting molecules involved in tumorigenesis and related signaling pathways in cancer cells, differentiate these cells from normal cells. Also, by targeting the tumor microenvironment, these drugs prevent tumor growth and cause anti-tumor immune attack and improve surveillance. The major types of targeted drugs are small molecule inhibitors and monoclonal antibodies [19].

Vascular Endothelial Growth Factor (VEGF) is the most important element involved in angiogenesis, which can affect the growth of colorectal cancer cells. Inhibition of VEGF may limit tumor growth and metastatic potential by affecting angiogenesis [20]. Bevacizumab is a monoclonal antibody against VEGF that prevents the growth of new blood vessels by inhibiting VEGF and thus inhibits tumor growth [21]. Instead of directly targeting cancer cells, bevacizumab targets the tumor microenvironment, which is characterized by important interactions between normal cells, extracellular matrix, and cancer cells [22]. This targeted drug is approved for first- and second-line treatment of metastatic CRC and shows significant efficacy when combined with conventional chemotherapy compared to chemotherapy alone [23]. Despite progress in understanding the mechanisms of resistance to antiangiogenic therapy, clinically effective approaches to overcome resistance to bevacizumab therapy are still limited [22].

Most of the time, the amount of EGFR protein is increased on the outer surface of colorectal cancer cells, which helps the cancer cells to grow. For this reason, targeted monoclonal antibodies that bind to EGFR, such as cetuximab, inhibit the proliferation of colorectal cancer cells by blocking intracellular signal transmission [24]. K-Ras gene polymorphism reflects changes in EGFR receptors and is related to the effectiveness of cetuximab. According to studies, metastatic colorectal cancer is often associated with genetic mutations such as K-Ras mutations. Currently, cetuximab combined with chemotherapy is the first line of attack for patients with wild-type K-Ras colorectal cancer [25].

In general, targeted therapies are designed to attack specific targets such as receptors or proteins on cancer cells. The goal of this method is to slow down or stop the growth of the tumor. This is done by attacking the genetic characteristics of the cell that lead to its growth and division. Also, these drugs may help with other cancer treatments, such as chemotherapy. Researchers are looking for specific gene mutations that can be targeted for colorectal cancer treatment.

## 3 Photothermal therapy

Recently, phototherapies such as photodynamic therapy (PDT) and PTT have become the focus of research on emerging treatments for a variety of cancers. In PTT, by activating the photothermal agent by light, light energy is converted into heat, and in PDT, by activating the photosynthesizer by light and with the support of dissolved oxygen, reactive oxygen species are produced [26]. Among the new cancer treatments, PTT is a non-invasive and advanced treatment method for efficient tumor ablation [27]. PTT in tumor ablation has advantages such as minimal invasiveness, less systemic toxicity, better tumor specificity, and temporal and spatial control of drug release [28]. The success of PTT primarily relies on the use of an optical absorbing agent, also known as a photosensitizer, which converts light energy into heat when stimulated by electromagnetic radiation such as microwaves, radio frequency, near-infrared (NIR), or visible light [29]. Through the absorption of light by PTT agents (PTAs), the electrons transition from the ground state to the excited state. These excited states are unstable and can return to lower energy levels by releasing heat. Consequently, this process leads to localized heating around the light-absorbing agents, resulting in the destruction of nearby cells or tissues [30].

Due to its ability to elevate the temperature of the targeted tissue, photothermal therapy PTT induces tumor cell death. Depending on the temperature reached, this cell death can occur through either necrosis or apoptosis. Necrosis is observed at temperatures equal to or greater than 50 °C, while apoptosis occurs within the temperature range of 43 °C to 50 °C. It is crucial in PTT to maintain an appropriate temperature range to ensure tissue destruction through apoptosis. This is because necrosis results in the release of intracellular contents, increasing the risk of cancer metastasis, whereas apoptosis does not affect the surrounding tissues. Biological tissues have the capacity to absorb varying levels of laser energy [31]. By applying thermal effects solely with near-infrared (NIR) light and a photothermal agent (PTA), cancer treatment can be performed with minimal damage to healthy surrounding tissues. NIR light, with a wavelength of 650–900 nm, is weakly absorbed by tissue and can penetrate deeper. Through proper design, selectivity can be improved by directing PTAs to the cancer site [28,32]. Researchers have developed various strategies to achieve the selective killing of tumor cells in PTT. For example, it is possible to raise the concentration of PTA in the target tumor tissue or use self-regulating PTAs. By increasing the concentration of PTA in the tumor site, it is possible to create a concentration discrepancy between the normal tissue and the tumor tissue. This will cause a choosy rise in the temperature at the tumor site. Self-regulating PTAs exhibit weaker photothermal conversion ability in normal tissue compared to tumor tissue [33].

The use of PTT alone, guided by imaging, or combined with existing treatments can inhibit cell migration by killing cancer cells in the primary tumor or nearby local metastasis [34]. The identification of the distribution of PTAs in vivo and the real-time monitoring of therapeutic efficacy are crucial in optimizing the practical application of PTT [35]. In imaging-guided PTT, targeted laser irradiation to the tumor minimizes side effects and by choosing the right time of radiation (when tumor retention by PTAs is maximized) treatment outcome is improved [36]. Furthermore, the development of nanoparticles that can generate heat under laser light radiation has garnered the attention of researchers in recent years due to their advantageous properties in this field [37].

Overall, PTT is one of the most promising non-invasive methods of colorectal cancer treatment, which requires temperature control in order to increase the temperature and maintain healthy tissue during the treatment process. Adding nanoparticles to the tissue causes a significant improvement in the treatment process.

## 4 Drug delivery systems for chemo-photothermal combination therapy

Chemotherapy is one of the effective and available methods of cancer treatment using some special drugs. However, drug resistance is one of the most important problems that can lead to the failure of this method by reducing the effectiveness of chemotherapy agents [38]. Although the effectiveness of chemotherapy in the clinical treatment of cancer can be increased to some extent by increasing the dosage of the drug, but it has very serious side effects. Although the effectiveness of chemotherapy in the clinical treatment of cancer can be increased to some extent by increasing the dosage of the drug, but it has very serious side effects. Therefore, to overcome these limitations, developing a drug delivery strategy with high drug loading capacity and intelligent release is of great importance for successful cancer treatment [39]. Recently, combination therapy combining chemotherapy with PTT has been rapidly developed to increase the therapeutic efficacy [40].

The combination of chemotherapy and PTT based on nanostructures have been able to increase the effectiveness of cancer treatment. Drugs based on nanomaterials can accumulate at the tumor site through active targeting (through surface conjugated molecules) or passive targeting (through increased permeability and retention effect) [40]. These nanostructures, in addition to penetrating the tissue system, facilitate the absorption of drugs by cells and increase the efficiency of drug delivery [41]. On the other hand, nanomaterial-mediated PTT targets the tumor and hyperthermia can be easily adjusted by controlling the intensity of the external energy source and time [42]. In general, the delivery of chemotherapy drugs simultaneously with PTT can produce a synergistic effect. Photothermal ablation combined with targeted drug delivery can effectively increase the therapeutic index and improve the treatment of multi-drug resistant cancers [43].

## 5 Application of nanoparticles in imaging and photothermal therapy

Nanotechnology has a high potential in the field of biomedicine, especially in relation to the diagnosis and treatment of CRC [44]. Nanoparticles possess several appealing attributes that render them attractive for cancer diagnosis and treatment, including their diminutive size, thermal stability, high affinity, remarkable solubility, and minimal off-target accumulation. Moreover, they demonstrate the ability to effectively penetrate dense tumor tissues, thereby enhancing control over biological distribution and therapeutic efficacy [45].

Nanoparticles used in cancer diagnosis either have intrinsic magnetic, optical, radioactive, or acoustic properties or carry molecules or nanoparticles that embody these properties. Furthermore, these characteristics can be tailored to the desired application by altering the physical properties of the nanoparticles [46]. The utilization of nanoparticle contrast agents has garnered significant attention in the realm of enhancing medical imaging techniques. This is due to their superior biocompatibility and reduced toxicity compared to conventional chemical agents [47]. Nanoparticles can be designed and adjusted in terms of biophysical interactions and biochemical interactions in such a way that they recognize distinct tumor microenvironments. Therefore, they can facilitate accurate and early cancer diagnosis by targeting the tumor and producing specific signals [48]. Additionally, numerous nanoparticles exhibit high absorption in the near-infrared (NIR) region, making them promising agents for deep photothermal therapy (PTT) of cancerous tissues. This is attributed to the fact that healthy tissues are incapable of absorbing light in this spectral region, and light scattering is minimized at longer wavelengths [49]. By modifying the synthesis of some nanostructures, an absorption peak can be adjusted in a very narrow range of wavelengths to improve the quality of the PTT effect. Too, by modifying the surface of many nanostructures, their biological stability can be modified, and toxicological concerns can be reduced [15].

Multifunctional nanoparticles with diagnostic and therapeutic functions have shown high potential for cancer treatment. The use of these nanoparticles in imaging-guided PTT is very significant because of the high selectivity and minimal invasiveness of PTT [50]. Multipurpose nanotechnology has been able to overcome some clinical effectiveness problems such as targeting problems, water solubility, stability and multidrug resistance. Surface modification of nanomaterials, in addition to protecting the internal materials against the harsh external environment, can act to apply new functions or control surface properties. For example, synthetic melanin nanoparticles with highly reactive chemical groups can be used to bind different functional components [51]. Despite the advances in the field of nanomaterial-based PTT, it still faces obstacles such as toxic side effects. Biomimetic nanomaterials synthesized using biomolecules with suitable functional properties have shown great potential to improve cancer treatment through PTT and photodynamic therapy (PDT). These synthesized nanomaterials have features such as suitable structure and morphology, regulated properties and functions, higher biocompatibility, and the ability to target tumor cells [52]. In the following, we will describe the function of metal nanoparticles, polymer nanoparticles, and carbon nanoparticles in CRC imaging-guided PTT.

### 5.1 Metal nanoparticles

#### 5.1.1 Gold nanoparticles

Gold nanoparticles (AuNPs) have been investigated as significant platforms in the field of diagnosis and therapy due to their great biocompatibility, simple synthesis, tunable optical properties, large surface area, and the possibility of being combined with other therapeutic agents through chemical bioconjugation [53]. Various forms of AuNPs have garnered considerable attention due to their unique optical-electronic properties. When exposed to light of a specific frequency, the free electrons on the metal surface become excited and reach their maximum oscillation amplitude, known as surface plasmon resonance (SPR). Through oscillatory non-radiative decay, light energy is converted into heat [54]. Consequently, AuNPs can exhibit photoacoustic and photothermal therapy (PTT) properties by absorbing light at specific wavelengths, making them suitable for medical imaging and cancer PTT applications. Modifying the shape and size of AuNPs can alter their photochemical activity and enable the absorption of different wavelengths of light in the near-infrared (NIR) spectrum [55]. The NIR region is ideal for AuNPs to penetrate deeper into tumor imaging and therapy. These nanoparticles, which are considered as contrast agents in photoacoustic imaging and PTT agents, can accumulate in cancerous tissue such as CRC through the enhanced permeability and retention (EPR) effect [56].

Gold nanorods (GNRs) are regarded as promising agents for PTT due to their ease of synthesis and rapid heat generation, as compared to spherical gold nanoparticles. Gournaris et al. developed NIR fluorophore-labeled GNRs for endoscopic near-infrared fluorescent (NIRF) imaging-guided PTT for the effective treatment of CRC. To assess the translational potential of this approach, the researchers intravenously injected bimodal GNRs into a mouse model of colon cancer. Immunofluorescent histological analysis revealed that the synthesized GNRs selectively accumulated in inflammatory macrophages in CRC, and provided ablation of inflammatory colonic polyps through endoscopic NIRF imaging. Therefore, the synthesized bimodal GNRs, in addition to NIRF imaging, showed effective photothermal effects under NIR laser irradiation. These effects can be used for the diagnosis and treatment of CRC and different inflammatory diseases [57].

**Fig. 2:**
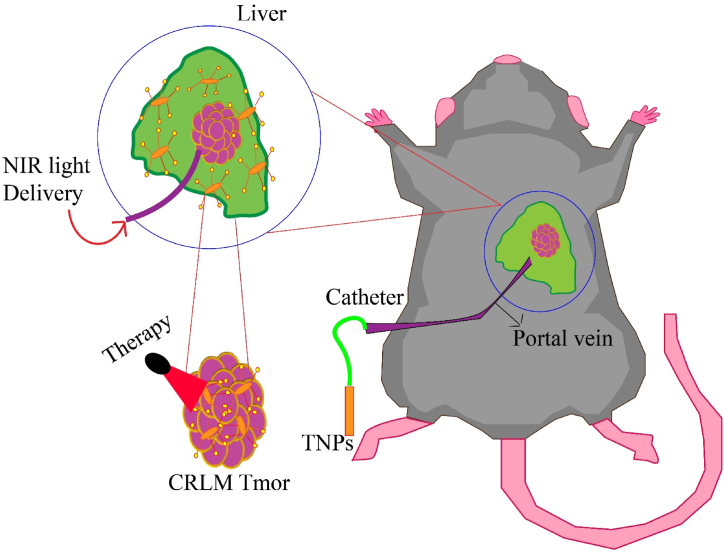
Schematic illustration of the selective delivery of TNPs through the hepatic portal vein and PTT using a catheter-based 808 nm NIR laser.

In a research study conducted by Parchur et al., optical/MR/X-ray contrast-enhanced theranostic nanoparticles (TNPs) were presented for the purpose of imaging-guided photothermal therapy (PTT) of colorectal liver metastasis (CRLM). The researchers synthesized gold nanorods (GNRs) coated with gadolinium oxide (Gd_2_O_3_) to achieve this objective. The primary aim of the study was to compare the absorption of TNPs through hepatic portal vein delivery with systemic administration in CRLM mice, and to investigate the feasibility of tumor PTT. MRI was utilized to demonstrate that the uptake of TNPs through hepatic delivery can double tumor contrast in comparison to systemic administration. Photothermal ablation was performed using an 808 nm light-carrying fiber under imaging guidance. The results of the histological analysis indicated that thermal damage was mostly confined to the tumor area, with minimal damage to adjacent liver tissue. In general, TNPs composed of GNRs containing Gd shells can be effectively used for interventional radiology-guided PTT at the site of CRLM (Fig. 2) [58].

Numerous studies have demonstrated the theranostic capabilities of Fe_2_O_3_@Au nanoparticles in vitro for cancer cells. In a conducted experiment, Abed et al. utilized Fe_2_O_3_@Au nanoparticles for in vivo MRI-guided targeted PTT of CRC. The gold shell's SPR property facilitates the realization of PTT, while the magnetic core allows for the utilization of the synthesized nanoplatforms as an MRI contrast agent. To assess these potentials, Fe_2_O_3_@Au nanoplatforms were administered to mice bearing CT29 colon tumors and subsequently exposed to a magnetic field. Following the tracking of the nanoparticles, the mice were subjected to NIR laser irradiation, and the efficacy of this strategy in inhibiting tumor growth was evaluated. The findings revealed that the prepared nanoplatform exhibits the potential to enhance the therapeutic response of PTT without causing harm to the surrounding healthy tissue. Consequently, Fe_2_O_3_@Au nanoparticles can be employed for targeted PTT of tumor cells under MRI guidance. However, further research is necessary to explore their future clinical applications [59].

Hyperthermia has been found to enhance the sensitivity of cancer cells to chemotherapy drugs and improve the efficacy of cancer treatment through various mechanisms. Considering this fact, Mirrahimi et al. developed a new nanocomposite consisting of alginate nanogel with AuNPs and cisplatin for a chemo-photothermal therapy combination. The researchers evaluated the antitumor efficacy of this nanocomposite on CT26 colorectal tumor model mice in the presence of 532 nm laser light. Based on the obtained results, this nano complex showed a developed chemotherapy effect compared to free cisplatin, and the treated tumors had a faster temperature increase under laser radiation. In addition, the measurement of tumor metabolism by PET imaging showed that this treatment method based on AuNPs could prevent cancer recurrence by significantly inhibiting tumor growth [60].

Due to its unique properties, AuNPs have been proposed as one of the most popular compounds in the field of imaging-guided PTT. Many methods of synthesis of AuNPs allow researchers to obtain them with desired architecture and characteristics. Also, the ability of these nanoparticles in functionalization and surface modification, availability, appropriate immunological properties increase their effectiveness in the medical field. The possible accumulation of AuNPs and its problem detection without the use of markers creates many obstacles. Especially the compounds used for coating and functionalizing AuNPs should be thoroughly tested due to possible side effects or toxicity in living organisms.

#### 5.1.2 Iron oxide nanoparticles

Iron oxide magnetic nanoparticles such as maghemite (γ-Fe_2_O_3_) and magnetite (Fe_3_O_4_) have emerged as hopeful theranostic agents due to their useful magnetic properties, biodegradability, and biocompatibility [61]. One of the applications of iron oxide nanoparticles (IONPs) in molecular imaging is to use them as targeted or non-targeted MRI contrast agents. In the MRI technique, the reduction of T1 and T2 relaxation time related to surrounding fluid protons causes positive and negative image contrast, respectively, and IONPs can improve MRI images by reducing relaxation time [62,63]. IONPs with higher saturation magnetization usually decrease the T2 relaxation time of the surrounding water and have better sensitivity for MRI detection. It has been shown that surface modification of IONPs and appropriate targeting ligands can increase the accumulation of IONPs in tumor tissues, while significantly reducing non-specific accumulation in the spleen and liver [64].

By subjecting IONPs to an external alternating magnetic field, heat can be generated as a form of energy. This phenomenon, known as magnetic hyperthermia, has the capability to induce thermal destruction of tumors [65]. However, the low molar absorption coefficient of IONPs in the NIR region necessitates their combination with other PAs. The utilization of nanocomposites comprising magnetic particles and PAs has garnered significant attention in the field of theranostics [66]. Therefore, IONPs have the potential to be used for imaging-guided PTT of cancer such as CRC by non-invasive detection and accumulation at the tumor site, so that the treatment can be applied exactly at the tumor site and at the right time.

In a study, Syu et al. created biocompatible iron oxide nanocrystals with excellent MR and NIR absorption properties through a facile ligand-assisted co-precipitation method. In addition to producing T2-weighted MR images, these nanocrystals provided good cancer photothermal ablation properties. These nanocrystals not only produced T2-weighted MR images but also exhibited remarkable cancer photothermal ablation properties. To enhance theranostic outcomes through magnetic field targeting at tumor sites, the synthesized nanocrystals were injected into HT-29 tumor-bearing mice and subjected to NIR laser irradiation. The results of PTT demonstrated significant changes in the intracellular protein of cancer cells and efficient disruption of their membranes, leading to the destruction of these cells. Furthermore, the proposed iron oxide nanocrystals exhibited high biocompatibility, excellent tumor accumulation ability, enhanced MR T2 contrast imaging, outstanding photothermal ablation, and minimal reticuloendothelial distribution. Therefore, iron oxide nanocrystals under NIR radiation hold great potential for MRI and PTT applications [67].

The core-shell iron oxide-gold (IO@Au) nanohybrid provides the possibility of location detection through MRI and magnetic tumor targeting and can activate PTT based on laser radiation. In order to develop a computational modeling method for predicting temperature distribution during PTT, Beik et al. conducted an experiment involving the injection of IO@Au nanoparticles into mice with CT26 colon tumors. The distribution of nanoparticles and the actual tumor geometry were determined using MR images of the mice, and the thermal damage to the tumor was assessed through histological analysis and monitoring of tumor growth. The obtained results show the potential of the built model to predict the temperature distribution, which can be improved by modifying the temperature parameters of the heat transfer to the tumor before the actual heating operation [68].

One of the attractive strategies for the preparation of theranostic nanoplatforms is the combination of biopolymers and inorganic nanoparticles. For example, Lin et al. proposed a human albumin/SPIO/IR70 (HISP) nanocomplex system for molecular imaging and PTT of colon cancer. After investigating the effect of polyethylene glycol (PEG) on HISP, the PEG-HISP nanocomplex was injected into CT26 tumor-bearing mice. The prepared nanoparticles exhibited T2-weighted MRI contrast, and clear images were obtained from the treated mice. Furthermore, these nanoparticles were internalized by cancer cells and induced cancer cell death through strong PTT upon short-term NIR radiation. Overall, the findings demonstrate that PEG-HISP is a potent and safe nanotherapeutic for cancer treatment [69].

According to research findings, polydopamine (PDA) has the ability to synergistically collaborate with Fe3O4 in order to reduce the energy requirements of laser treatment and enhance thermal effects. In a study, Fan et al. presented a targeted Fe_3_O_4_@PDA-PEG-cRGD-DOX nanocomposite to integrate tumor diagnosis and treatment. In vitro and in vivo studies showed their high capacity to target tumor cells. In addition to having thermal stability and photothermal conversion effectiveness, these nanoparticles released DOX in response to pH in a mildly acidic environment. Additionally, the synthesized nanoparticles displayed excellent MRI contrast in nude mice with colon cancer and effectively suppressed tumor growth when exposed to NIR radiation. In summary, the multifunctional Fe_3_O_4_@PDA-PEG-cRGD-DOX nanoparticles can be used as an effective carrier for tumor diagnosis and the combination of PTT and chemotherapy [70].

According to studies, IONPs have a high potential as a factor in the diagnosis and PTT of colorectal cancer. These nanoparticles can not only be used as drug carriers, but can also be directed to a specific area of the body through an external magnetic field and show various applications in the field of biomedicine. Despite some common methods for the synthesis of these nanoparticles, the methods used for their preparation still need to be improved in order to achieve desirable physicochemical and biological properties. Therefore, the functionalization of IONPs is the most important step to avoid the effects of toxic effects in biomedical applications.

#### 5.1.3 Other metal nanoparticles

Silver nanoparticles (AgNPs) have been widely investigated in the field of biomedicine due to their unique physical and chemical properties, such as ease of synthesis, high thermal conductivity, chemical stability, antibacterial ability, and promising self-healing potential [71]. The functionalization of AgNPs with diverse molecules can raise their half-life time in vivo. Additionally, by adjusting the optical properties of these nanoparticles, AgNPs can selectively absorb light at specific wavelengths, making them useful in imaging and photothermal therapy (PTT) with improved penetration and the ability to track their location [72]. AgNPs with strong optical absorption and scattering in the NIR and visible regions are used for non-invasive diagnostic purposes such as photoacoustic microscopy, dark field microscopy, and surface-enhanced Raman scattering (SERS) [73]. These nanoparticles, which have good absorption in the NIR region, exert their photothermal effect through plasma resonance effects on the particle surface. When light is irradiated onto AgNPs, it intensifies the presence of free electrons on the nanoparticle surface, resulting in the generation of an exothermic electron gas. The transfer of energy to the surrounding environment subsequently leads to an increase in temperature [74]. Overall, the enhanced functionalities of AgNPs motivate researchers to design nanocomposite platforms with both photothermal therapy and imaging capabilities. The development of smart integration strategies can increase the bioefficacy of these nanoparticles in imaging-guided PTT to eradicate tumor cells such as CRC [75].

**Fig. 3:**
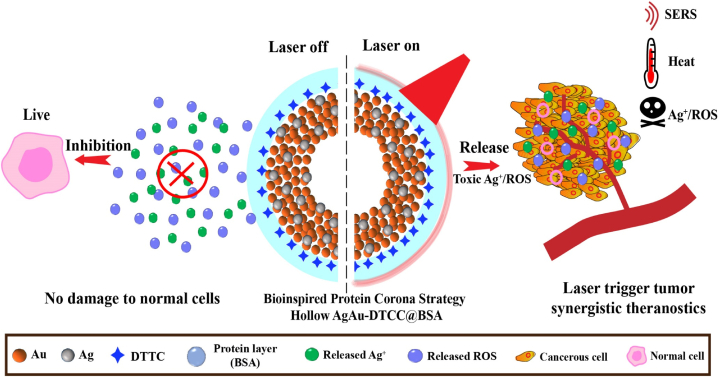
Schematic illustration of the use of hollow AgAu-DTTC-BSA nanoshells for guided PTT imaging of CRC cells.

He et al. presented a protein strategy that is both simple and versatile. They introduced Raman-labeled hollow Ag–Au nanoshells, which were protected by a bovine serum albumin (BSA) layer, for the purpose of detecting and performing PTT on colon cancer. These hollow nanoshells were designed to prevent the release of Ag ions and reactive oxygen species (ROS), thereby reducing cytotoxicity to normal cells and tissues after being injected into CT26 colon tumor-bearing mouse models. Importantly, these nanoparticles have the ability to reactivate the production of Ag ions and ROS specifically at the tumor site when exposed to NIR radiation. Also, these hollow Ag–Au nanoshells can produce strong LSPR and show efficient and stable PTT effect and increase SERS contrast under laser irradiation. In conclusion, this composite nanoplatform is promising for imaging-guided cancer PTT (Fig. 3) [75].

To overcome the challenge of efficient tumor targeting while reducing side effects in PTT, Wang et al. reported multifunctional phage protein-modified Au@Ag heterogeneous nanorods (PMHNRs) for targeted detection and PTT of CRC cells. The study involved the isolation of fusion phage proteins from the specific phage of SW620 cells, which were then exposed to the surface of PMHNRs due to their unique bipolar properties. Fluorescent imaging revealed that the phage fusion proteins facilitated targeted PTT specificity by guiding the nanostructure into cancer cells. Furthermore, under 10 min of 808 nm laser light with a power of 4 W/cm2, the assembled Au@Ag nanorods were able to effectively eliminate SW620 cells. Thus, the PMHNRs, which are introduced for the first time in this paper, hold great potential as an ideal agent for simultaneous PTT with tumor imaging [76].

Manganese oxide nanoparticles such as MnO_2_ have been investigated as promising contrast agents in cancer theranostic. Mn^2+^ particles with T1 contrast effect increase the signal intensity of T1-weighted images [77]. The three-dimensional orbit of Mn^2+^ containing five unpaired electrons generates a significant magnetic moment, resulting in strong MRI properties for MNO_2_ nanoparticles by relaxing the nearby water proton. These nanoparticles possess the ability to respond to the tumor microenvironment (TME), including pH, glutathione, or H_2_O_2_, thereby enabling tailored treatment outcomes for tumor tissues [78]. In addition, the solubility of Mn^2+^ ions in water causes their hydrodynamic excretion, which can prevent long-term toxicity and unnecessary accumulation in the body [79]. Studies have shown that some MnO_2_ nanoparticles in high volumes can have enough energy to destroy the tumor with the efficiency of photothermal conversion. However, the antitumor efficiency of these nanoplatforms at low concentrations is not satisfactory. Many attempts have been made to combine photothermal conversion agents such as polydopamine (PDA) with MnO_2_ nanomaterials to facilitate tumor ablation in combination with PTT. It is hypothesized that PDA may overcome the shortcomings of these nanoplatforms due to strong absorption in the NIR region and act as a potential photothermal agent to increase PTT efficiency [80,81].

Ultrathin MnO_2_ nanosheets (ut-MnO_2_) have shown great superiority for cancer theranostic usage due to their inherent structural advantages. In a study, Sun et al., with a simple redox strategy, synthesized multifunctional nanoflowers by controllable growth of ut-MnO_2_ nanosheets on PDA nanospheres. Upon loading these nanoflowers with methylene blue (MB), the PDA@ut-MnO2/MB NFs nanoplatform was obtained, exhibiting high photothermal conversion efficiency for CRC PTT. The prepared nanoflowers were capable of modulating the tumor microenvironment and enhancing the efficacy of photodynamic therapy (PDT) by converting endogenous acidic H2O2 to O2 and reducing GSH. In addition, the generation of Mn^2+^ ions by the decomposition of ut-MnO_2_ nanosheets acted as MRI contrast agents for precise treatment guidance. The results of using PDA@ut-MnO_2_/MB NFs in mice bearing HCT116 colorectal tumors have shown almost complete suppression of tumor growth following the synergistic effect of MRI-guided PTT/PDT [82].

Owing to its potent ability to eliminate cancer cells and stimulate the immune system, mild PTT has exhibited significant potential in sensitizing tumors to immune checkpoint inhibition. Li et al. have developed MnO_2_@mPDA-PEG nanomedicine for mild PTT of CRC by integrating MnO_2_ into PEGylated-mesoporous polydopamine (mPDA) nanoparticles. The presence of PEG in these nanoparticles increased the biostability of the synthesized nanomedicines and caused their accumulation in colorectal tumors. In addition to acting as an MRI contrast agent, the released Mn^2+^ ions were able to enhance the mild immune response induced by PTT, such as the maturation of bone marrow-derived dendritic cells (BMDCs). Overall, MnO_2_@mPDA-PEG nanoparticles hold great promise as a therapeutic and imaging nanomedicine to enhance systemic antitumor immunity [83].

Nanostructures based on molybdenum disulfide (MoS_2_) have garnered significant attention due to their advantageous properties, including mechanical stability, adjustable electrical properties, and photochemical reactivity [84]. Various types of theranostic agents based on MoS_2_ nanoparticles have been developed and successfully investigated in biomedical applications such as cancer diagnosis and PTT [85]. These nanoparticles possess remarkable flexibility as they can easily be combined with other nanomaterials or functionalized with biological molecules. The extensive specific surface area of MoS_2_ enables the integration of various molecules through covalent and non-covalent interactions, thereby constructing robust imaging platforms. Furthermore, the high absorption rate of these nanoparticles across a broad range of wavelengths enhances their applicability in PTT [86]. The functionalization process of MoS_2_ nanoparticles can help their effectiveness and selective treatment in cancer cells. For example, local hyperthermia controlled by NIR can be used alone or in combination with various other methods to treat cancer [87].

The augmentation of MoS2 nanoparticles with thiolated hyaluronic acid (HA) can lead to an increase in physiological stability, biocompatibility, and tumor-targeting efficiency. This is due to the fact that HA facilitates the delivery of MoS2 to tumor cells through the HA receptor. Shin et al. successfully reported multifunctional HA-MoS_2_ conjugates for multimodal bioimaging and PTT of CRC. After absorption of conjugates prepared by HA receptors on tumor cells, their disulfide bonds are destroyed, and the accumulation of MoS_2_ nanoparticles in the cytoplasm enhances the optical signal and the photothermal effect in the tumor cells. In this study, the efficiency of multimodal imaging and PTT of HA-MoS_2_ conjugates was observed in nude mice inoculated with HCT116 CRC cells under pulsed and continuous NIR radiation. As a result, HA-MoS_2_ conjugates can be developed as a cancer theranostic nanoplatform [88].

Polyvinylpyrrolidone (PVP) surfactant, in addition to controlling corn size, also causes the colloidal stability of products and can act as a template to guide the growth of nanosheets. In a study, Zhao et al. developed a PVP-assisted one-pot hydrothermal approach for the simultaneous synthesis and surface modification of two-dimensional MoS_2_ nanosheets. The resulting ultrasmall MoS_2_-PVP nanosheets exhibited high compatibility and photothermal conversion performance, and were utilized for PTT of mice with HT29 CRC cells. These nanosheets demonstrated photoacoustic imaging and photothermal conversion performance due to their NIR absorption. These findings underscore the therapeutic potential of MoS_2_-based nanomaterials and may inspire further research aimed at designing nucleus-targeting tumor PTT systems [89].

As mentioned, metal nanoparticles show unique optical, electrical and magnetic properties due to their very high surface-to-volume ratio and suitable intrinsic properties, and their properties can change depending on the size of the nanoparticles. Metal nanoparticles have a high potential in cancer treatment, tumor targeting in tissues, targeted drug delivery, biological imaging, gene therapy and other diagnostic and therapeutic sectors. Table 1. Some examples of metal nanoparticles for imaging-guided PTT of colorectal cancer.

**Table 1:** Some examples of metal nanoparticles for imaging-guided PTT of colorectal cancer.

Types of nanoparticles	Size	Imaging	Laser wavelength (nm)	Temperature	Colorectal cancer cell line	In vitro/In vivo	Ref.
GNRs	12.7 ± 3.4 nm in width and 47 ± 9.3 nm in length	NIRF endoscopic imaging	808	ΔT∼ 20 °C	–	In vivo	[57]
Au@Gd_2_O_3_ NPs	∼75 nm in diameter	MRI and X-ray imaging	808	ΔT∼ 19.5 °C	CC-531	In vitro and in vivo	[58]
Fe_2_O_3_@Au NPs	22 nm in diameter and ∼5 nm in thickness	MRI	808	ΔT∼ 12 °C	CT26	In vivo	[59]
Alginate nanogel co-loaded AuNPs	20–80 nm in the hydrodynamic diameter	(2-deoxy-2-[18F] fluoro-d-glucose)- PET	532	T_max_ = 58.55 °C	CT26	In vivo	[60]
AuPEI nanocomposite	10.8 nm	Photoacoustic imaging	808	T_max_ > 50 °C	CT26	In vitro and in vivo	[90]
Fe_3_O_4_@RhB/SiO_2_@Au nanoparticles	365 nm in diameter	MRI and optical imaging	808	–	SW620	In vitro	[91]
Iron Oxide Nanocrystals	182.7 nm in hydrodynamic size	MRI	808	T_max_ = 51 °C	HT-29	In vitro	[67]
IO@Au nanoparticles	20 nm in diameter, and ∼3 nm in thickness	MRI	808	T_max_ = 48.6 °C	CT26	In vivo	[68]
PEGylated albumin/IR780/Iron oxide nanocomplexes	146.3 nm and 173.7 nm	MRI	808	ΔT∼14 °C	CT26	In vitro and in vivo	[69]
Fe_3_O_4_@PDA-PEG-cRGD-DOX nanoparticles	275.4 nm	MRI	808	T_max_ = 45.5 °C	HCT116	In vitro and in vivo	[70]
Iron oxide-hydroxide nanospindles	around 10 × 40 nm	MRI	808	T_max_≈ 59 °C	CT26	In vitro and in vivo	[92]
Gold-iron oxide hybrid nanoparticles	6–18 nm	MRI	808	T_max_ = 42–45 °C	SW1222 and HT29	In vitro and in vivo	[93]
hollow AgAu-DTTC-BSA nanoparticles	∼85 nm in hydrodynamic size	SERS imaging	785 and 808	T_max_ ∼52 °C	CT26	In vitro and in vivo	[75]
Phage protein-modified Au@Ag heterogeneous nanorods	220 nm in length and 70 nm in width	Fluorescence and optical imaging	808	–	SW620	In vitro	[76]
PDA@ut-MnO_2_/MB nanoflowers	82 ± 5 nm in diameter	MRI	808 and 650	T_max_ = 52 °C	HCT116	In vitro and in vivo	[82]
MnO_2_@MPDA-PEG nanoparticles	∼180 nm in hydrodynamic size	MRI	808	T_max_ = 43–45 °C	CT26	In vitro and in vivo	[83]
HA-MoS_2_ conjugates	≈200 ± 75.31 nm	Photoacoustic and fluorescence imaging	808	ΔT > 25 °C	HCT116	In vitro and in vivo	[88]
MoS_2_-PVP nanosheets	21.4 ± 4.4 nm	Photoacoustic imaging	808	ΔT = 23.5 °C (after 300 s of iradiation)	HT29	In vitro and in vivo	[89]

### 5.2 Polymeric nanoparticles

Polymeric nanoparticles have attracted widespread attention in the field of theranostics due to their advantages such as excellent biocompatibility, tunable design, optical stability, and ease of modification [94]. These nanoparticles possess a high capacity for carrying diagnostic molecules, making them valuable cancer diagnostic tools capable of delivering imaging agents directly to the tumor site [95]. In addition, polymer-based PTAs can effectively induce local hyperthermia by converting light energy into heat. By rationally designing polymer nanoparticles and modifying their structure with amphiphilic polymers, it is possible to reduce the laser dose and intensity to produce sufficient heat [96]. Natural source-derived polymers, such as albumin and chitosan, have received considerable attention in the development of biocompatible polymer nanoparticles. Additionally, a wide range of synthetic polymers with high biodegradability and biocompatibility can be utilized to formulate polymeric nanoparticles [97]. Therefore, polymer-based nanoparticles have attracted the attention of researchers for the development of cancer theranostic platforms.

#### 5.2.1 Natural polymer nanoparticles

Natural polymers are generally produced by microorganisms, animals, and plants and are divided into three main categories: polysaccharides, polypeptides, and polyesters [98]. These polymers, which have inherent properties related to biodegradation, have been studied in the field of nanomedicine for a long time. The distinctive biological characteristics of natural polymers, such as the presence of tumor-specific ligands, mucus adhesion, or rapid degradation by tumor enzymes, make them well-suited for the development of nanoparticles for effective cancer diagnosis and treatment [99]. Structures similar to natural polymers with the extracellular matrix (ECM) of native tissues increase cell attachment, improve cell structure, and avoid immune reactions. However, some of these polymers have limitations, such as poor mechanical strength, which needs to be modified to obtain suitable mechanical properties [100].

Hyaluronic acid (HA), a natural polysaccharide, serves as the primary constituent of the extracellular matrix (ECM). Various HA receptors, including cluster of differentiation 44 (CD44) found on cancer cells, are known to facilitate selective targeting of tumors [101]. Cheng et al. developed a nanoplatform that responds to hydrogen sulfide (H_2_S) and exhibits therapeutic and diagnostic properties for colon cancer. This nanoplatform, known as HA-modified bismuth-doped cuprous oxide nanoparticles (Bi: Cu_2_O@HA NPs), enhances the accumulation of synthesized nanoparticles specifically at colon cancer tumor sites by virtue of HA's targeting ability. While doping with Bi, in addition to increasing the photothermal performance of Cu_2_S stimulated with H_2_S, it acts as a CT agent for tumor imaging. The findings of this study demonstrate that H_2_S-stimulated Bi: Cu_2_O@HA NPs exhibit remarkable photothermal effects in the treatment of colon cancer, while avoiding toxic side effects on healthy tissues. Overall, the newly synthesized nanoparticles showed good therapeutic effects for tumor targeting and PTT, which provides new insights and strategies for colon cancer treatment [102].

**Fig. 4:**
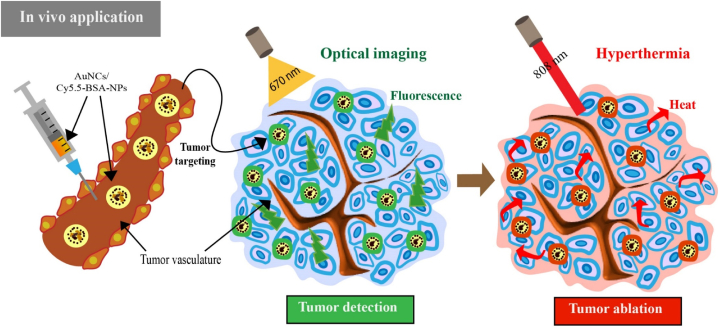
Schematic illustration of in vivo application of AuNCs/Cy5.5-BSA-NPs with fluorescence intensity and hyperthermal activity for tumor detection and ablation.

Protein-based biocompatible nanocarriers have been chosen as building blocks for the construction of multifunctional theranostic platforms. In particular, albumin has shown great promise in the development of imaging and therapeutic nanoagents [103]. In accordance with these attributes, Park et al. devised and synthesized hybrid albumin nanoparticles incorporating gold nanoclusters (AuNCs) to enable visualization and photoablation of colon cancer in response to NIR light. In this study, albumin was considered a critical factor in the distance between AuNC and Cy5.5 dye, and a series of formulated AuNCs was produced. Then, to maintain the fluorescence intensity while maximizing the hyperthermal effect, the formula of hybrid nanoparticles was optimized by adjusting the distance between the particles. The outcomes revealed that AuNCs/Cy5.5-BSA, synthesized in response to NIR light, exhibited a favorable hyperthermal effect in suppressing colon cancer tumors in HCT116-bearing mice, while simultaneously visualizing the tumor sites and maintaining fluorescence intensity. Consequently, these researchers successfully achieved hyperthermia and fluorescence emission by optimizing the particle distance in the proposed nanoplatforms (Fig. 4) [104].

Chitosan oligosaccharide lactate (COL) is a natural carbohydrate polymer that is suitable for biomedical applications. When combined with near-infrared (NIR) fluorophores, COL can function as an imaging and photothermal agent. In a study, Lee et al. showed that COL-ZW consisting of natural COL and biocompatible fluorophore ZW800-1 could be a theranostic agent for fluorescence-guided PTT in the treatment of CRC. During the study, HT-29 tumor-bearing mice were subjected to 808 nm NIR radiation, resulting in an increase in the temperature of COL-ZW. Consequently, the volume of the targeted tumor decreased within a week following treatment. Notably, no treatment-induced toxicity or tumor recurrence was observed after administering a single dose of the proposed conjugate with laser irradiation. So, COL-ZW as a reliable and safe theranostic agent, has great potential for the improvement of next-generation cancer therapies [105].

Nowadays, due to the many problems caused by the side effects of some nanoparticles, researchers have been led to use natural polymers. Natural polymers have properties such as chemical changeability, biocompatibility and biodegradability. Plant-based polymers can present some challenges, such as synthesis in small quantities or mixtures with complex structures, which may lead to additional costs in the isolation process.

#### 5.2.2 Synthetic polymer nanoparticles

In comparison to natural polymers, synthetic polymers offer numerous advantages, including ease of synthesis, minimal variation between categories, and a low likelihood of microbial contamination. Furthermore, their ability to exhibit sufficient functional groups and surface area allows for their combination with targeting or imaging agents [99]. Synthetic polymers are designed and synthesized with a diversity of structures and opportune physical and chemical properties and are used in a wide range of biomedical utilization, such as cancer diagnosis and treatment [106]. Among the most commonly utilized synthetic polymers is PEG, which not only demonstrates exceptional stability and solubility in water but also possesses low immunogenicity and antigenicity. In addition, polylactic acid (PLA) and polylactic-*co*-glycolic acid (PLGA), which undergo hydrolysis in living organisms and have good biocompatibility, are receiving the attention of researchers [107].

In a study, Xi et al. synthesized PLGA nanoparticles loaded with the chemotherapeutic drug doxorubicin (DOX) as a substrate for synergistic PTT-chemotherapy of colon cancer. Then these nanoparticles were modified with polydopamine (PDA), and Mn^2+^ ions were coordinated on them. Mn^2+^-PDA@DOX/PLGA nanoparticles were able to destroy the tumor directly by thermal energy deposition in a mouse model and enhance cancer treatment by thermal delivery of DOX. In addition, Mn^2+^ coordination enabled high-relativistic MRI. Overall, these proposed PLGA-based nanoparticles have great potential in clinical applications as a smart therapeutic agent due to their imaging and tumor growth inhibition properties [108].

In another study, Mohammadi Gazestani et al. conducted a study involving the development of magnetite nanographene oxide (NGO-SPION) coated with PLGA and loaded with 5-FU. The purpose of this study was to combine (PTT and chemotherapy for the treatment of colon cancer. The nanoparticles, designated as NGO-SPION-PLGA-5-FU, were found to possess favorable pharmacokinetic properties, including a prolonged half-life of the drug and sustained release in rabbit plasma. Furthermore, the administration of a single injection of these nanoparticles, coupled with 808 nm NIR laser irradiation for a duration of 3 min, effectively suppressed tumor growth in comparison to the use of 5-FU alone. The efficacy of tumor targeting by the nanoparticles was confirmed through the use of MRI imaging. Consequently, the synthesized nanoparticles, which possess magnetic targeting properties, MRI capabilities, and demonstrate excellent PTT effectiveness, exhibit significant potential for applications in cancer theranostics [109].

PHEA is a biocompatible synthetic polymer derived from polysuccinimide, which is usually used for various drug delivery applications. Puleio et al. prepared gold nanorods (AuNRs) coated with PHEA containing lipoic acid (LA) molecules, PEG chains, and folate. After loading the anti-neoplastic drug irinotecan (Iri), these researchers investigated the effect of the prepared nanosystem on PTT and chemotherapy for colon cancer. Biodistribution data revealed that the polymer-coated nanorods exhibited a preference for accumulating at the tumor site. Following laser treatment, these nanostructures were able to effectively reduce tumor growth. When administered intratumorally to a mouse xenograft model of colon cancer, complete eradication of the cancer was observed. To further validate the efficacy of PTT using laser, the tumor volume was qualitatively monitored through MRI, which demonstrated a reduction in tumor size. Considering the excellent stability of PHEA-LA-Fol-AuNRs/Iri in aqueous environments, the capacity to reach the tumor site, and their effectiveness in vivo conditions, this nanosystem may be suggested as an effective tool for PTT and chemotherapy of colon cancer [110].

By using appropriate biopolymers and selective additives, synthetic polymers with desired properties can be synthesized. The poor dispersion and mechanical behavior of composite polymers limit the synthesis of biocomposites, so their analysis and development are necessary to improve their use in biomedicine. Table 2. Some examples of polymer nanoparticles for imaging-guided PTT of colorectal cancer.

**Table 2:** Some examples of polymer nanoparticles for imaging-guided PTT of colorectal cancer.

Types of nanoparticles	Size	Imaging	Laser wavelength (nm)	Temperature	Colorectal cancer cell line	In vitro/In vivo	Ref.
Bi:Cu_2_O@HA nanoparticles	63.09 nm	CT imaging	808	T_max_ = 47.23 °C	CT26	In vitro and in vivo	[102]
AuNCs/BSA nanoparticles	∼150 nm	Photoacoustic and fluorescence imaging	808	T_max_ >50 °C	HCT116	In vitro and in vivo	[104]
Ellagic acid Fe@BSA nanoparticles	13.84 ± 2.53 nm in diameter	MRI	808	T_max_ = 41 °C	HCT116	In vitro and in vivo	[111]
chitosan oligosaccharide lactate-ZW800-1	1–1.5 nm	NIR fluorescence imaging	808	T_max_ ∼ 62.3 °C	HT-29	In vitro and in vivo	[105]
Mn^2+^-PDA@DOX/PLGA nanoparticles	∼200 nm in hydrodynamic size	MRI	808	T_max_ = 55 °C	CT26	In vitro and in vivo	[108]
NGO-SPION-PLGA-5-Fu	72.9 nm	MRI	808	T_max_ = 43.3 ± 0.52 °C	CT26	In vitro and in vivo	[109]
PHEA-LA-Fol-AuNRs/Iri	about 45 × 15 nm	MRI	810	T_max_ = 63 °C	HCT116	In vitro and in vivo	[110]

### 5.3 Carbon nanoparticles

Carbon nanoparticles possess unique structures and physicochemical properties that make them highly valuable in the field of biomedicine [112]. Most allotrope forms of carbon appear to have negligible toxicity and are widely biocompatible with various agents, making it an ideal nanomaterial for research in various applications [113]. The modification of these nanoparticles' surfaces with functional groups presents an opportunity to optimize their properties, and their high surface area, favorable electrical and mechanical properties make them suitable for theranostic applications. Crucially, their aqueous stability and interaction with cells and tissues ensure the biological safety of these nanoparticles [114]. Many carbon nanomaterials such as carbon nanotubes (CNTs), graphene derivatives, carbon dots (C-dots), and nano-diamonds (NDs) with interesting optical properties such as intrinsic fluorescence are useful contrast agents for optical imaging and sensing. In addition, some carbon nanomaterials with strong light absorption in the NIR region are also useful for the photothermal ablation of cancer [115].

The GE11 peptide is recognized as an effective ligand for the EGFR and is utilized as a targeting agent. Accordingly, Qiu et al. modified graphene oxide (GO)-based nanosheets loaded with chemotherapy drug 5-FU with GE11 and investigated their efficacy for combined PTT/chemotherapy of CRC. Following the administration of the prepared 5-FU/GO-PEG-GE11 nanocomposites into mice bearing CRC, the treatment groups were subjected to 808 nm NIR laser radiation. The results demonstrated a synergistic antitumor effect, with a tumor inhibition rate of 90 % in the CRC mouse model. Therefore, this GO-based multimodal nanoplatform holds great promise for PTT-based antitumor therapy [116].

Fullerene (C60) is one of the allotropes of carbon that has attracted a lot of attention and is widely studied [117]. Fullerene molecules within fullerene nanocrystals (FNCs) possess exceptional crystallinity and offer the potential to enhance the photoacoustic signal and convert light energy into heat. These features encouraged Kawasaki et al. to develop theranostic nanomaterials based on FNCs combined with GNPs for photoacoustic imaging and PTT of colon cancer. Through the exposure of prepared nanoparticles to laser light, these particles effectively convert light into ultrasound waves and heat energy, thereby obliterating cancer cells under laboratory conditions. Furthermore, the outcomes of photoacoustic imaging in tumor xenograft mice have demonstrated the utility of this system as a contrast agent for cancer diagnosis [118].

Chitosan biopolymer can act as a reducing and stabilizing agent for reduced graphene oxide (rGO) nanoparticles. In a study, Zaharie-Butucel et al. designed a Chit-rGO nanosystem carrying IR820 dye and DOX to integrate chemotherapy, PTT, PDT, and traceable optical properties. Initially, the researchers examined the ability of the prepared nanoplatforms to generate oxygen and heat when exposed to 785 nm NIR radiation in an aqueous solution. Subsequently, the anticancer activity of these nanoparticles against mouse colon cancer cells was evaluated. The Chit-rGO-IR820-DOX nanosystem, in conjunction with PDT, PTT, and chemotherapy, exhibited synergistic effects against C26 cancer cells. These researchers found that the most efficient therapeutic effect on these cancer cells is produced by the low DOX/high IR820 platform. Also, in addition to visualizing rGO inside cancer cells, confocal Raman microscopy facilitates the localization and accumulation of DOX in these cells. As a result, this class of nanocarriers is promising for use in the treatment of cancers [119].

Carbon nanostructures with interesting physical and chemical properties are highly effective in various medical fields such as PTT guided imaging of colorectal cancer. According to the characteristics of these nanoparticles, researchers can improve them and meet their needs. However, due to the fact that there have been fewer studies regarding the investigation of negative effects, it is suggested to pay more attention to the harmful effects related to carbon nanostructures in future studies and researches. Table 3. Some examples of carbon nanoparticles for imaging-guided PTT of colorectal cancer.

**Table 3:** Some examples of carbon nanoparticles for imaging-guided PTT of colorectal cancer.

Types of nanoparticles	Size	Imaging	Laser wavelength (nm)	Temperature	Colorectal cancer cell line	In vitro/In vivo	Ref.
5-FU/GO-PEG-GE11 nanocomposite	245 ± 4 nm in hydrodynamic size	Fluorescence imaging	808	T_max_ = 63 °C	HCT-116	In vitro and in vivo	[116]
FNCs-GNPs	98 nm in hydrodynamic diameter	Photoacoustic imaging	680	ΔT = 10 °C	Colon26	In vitro and in vivo	[118]
chit-rGO-IR820-DOX nanoplatform	220 nm in hydrodynamic diameter	Raman imaging	785	ΔT = 20.6 °C	C26	In vitro	[119]

## 6 Conclusion

Discovering effective methods of cancer treatment that yield optimal therapeutic outcomes while minimizing invasiveness is a paramount challenge that has consistently captivated the attention of researchers. Swift cancer diagnosis and early-stage treatment play a pivotal role in curtailing treatment expenses and expediting patient recovery. Among the various cancer treatment modalities, PTT stands out as a particularly promising approach. This is due to the fact that the wavelength employed in PTT coincides with the lowest light absorption by the most significant light-absorbing agents within the body. Synergistic treatments incorporating PTT have demonstrated remarkable efficacy in combating cancer. To ensure the successful implementation of the high-efficiency PTT method, the presence of a light-to-heat conversion agent is indispensable. Consequently, in this method, the efficiency of photothermal conversion agents must be taken into account, in addition to the intensity and duration of laser radiation. This consideration is crucial to enable deep penetration into tumor tissues while minimizing harm to healthy tissues.

Recently, nanotechnology has opened new windows for researchers by introducing new nanostructures that have interesting features in the fields of diagnosis and PTT. This is because nanoparticles with high absorption of light are the most effective for converting light into heat. In this article, we tried to introduce the use of different nanoparticles (metal, polymer, and carbon) as imaging-guided PTT agents for the treatment of CRC. Furthermore, we have provided a comprehensive overview of multifunctional synergistic therapies that employ these nanoparticles to enhance their therapeutic effectiveness. These nanoparticles have demonstrated the ability to enhance the efficacy of PTT in CRC treatment by specifically targeting the tumor and deeply penetrating the tumor tissues, particularly when guided by imaging techniques. In addition to accurately identifying the location of cancer cells, imaging techniques also enable the monitoring of the efficacy of photothermal therapy. The small size of nanoparticles allows them to accumulate inside target tissues and organs by penetrating through small capillaries, and the use of biodegradable nanoparticles can be used for the controlled release of encapsulated biomolecules. But nanotechnology also has disadvantages such as air pollution, higher development costs, endangering living organisms, and lung damage by inhaling air-containing nanoparticles [120]. Due to their small size, nanoparticles may penetrate the physiological barriers of living organisms and cause harmful biological reactions. In this way, when nanoparticles enter the body through the skin, lungs and intestinal tract, they can be toxic to the brain and cause heart problems and lung inflammation. Also, these particles may cause permanent cell damage through oxidative stress. Therefore, for clinical use, it must be proven that these nanoparticles are cleared from the body after treatment without side effects [121]. In addition, nanoparticles can have a negative impact on the environment. In this way, the disposal of nanoparticles in the environment can have a negative impact on the health of marine organisms and increase their cellular toxicity. Accumulation of nanoparticles in the soil can also reduce the rate of photosynthesis of plants [121]. So, in order to fully understand the impact of pollution on the environment, more studies should be done and until their effects are fully determined, the necessary precautions should be taken when using nanoparticles.

Despite the numerous advancements made in the development of photothermal therapy (PTT) for the treatment of colorectal cancer (CRC), there are still several challenges that need to be addressed. These challenges include the toxicity of photothermal agents and the issue of thermal resistance. Furthermore, the current results obtained from PTT studies are limited to preclinical models. However, it is highly probable that future research will enhance the potential of nanomaterials in improving cancer PTT during clinical phases. This article aims to provide researchers with a comprehensive understanding of the application of nanoparticles in imaging-guided PTT for CRC treatment. With this knowledge, we anticipate favorable outcomes in the clinical application of this treatment method in the near future.

## Data availability statement

No data was used for the research described in the article.

## CRediT authorship contribution statement

**Omid Rahbar Farzam:** Writing – review & editing, Writing – original draft, Investigation. **Niloofar Mehran:** Writing – review & editing. **Farzaneh Bilan:** Writing – review & editing. **Ehsan Aghajani:** Writing – review & editing. **Reza Dabbaghipour:** Writing – review & editing. **Ghazale Asemani Shahgoli:** Writing – review & editing. **Behzad Baradaran:** Writing – review & editing, Supervision, Project administration.

## Declaration of competing interest

The authors declare that they have no known competing financial interests or personal relationships that could have appeared to influence the work reported in this paper.
